# TaqMan Probe-Based Quantitative Real-Time PCR to Detect *Panax notoginseng* in Traditional Chinese Patent Medicines

**DOI:** 10.3389/fphar.2022.828948

**Published:** 2022-05-24

**Authors:** Qian Lou, Tianyi Xin, Wenjie Xu, Ranjun Li, Jingyuan Song

**Affiliations:** ^1^ Key Lab of Chinese Medicine Resources Conservation, State Administration of Traditional Chinese Medicine of the People’s Republic of China, Institute of Medicinal Plant Development, Chinese Academy of Medical Sciences and Peking Union Medical College, Beijing, China; ^2^ Engineering Research Center of Chinese Medicine Resource, Ministry of Education, Beijing, China; ^3^ Yunnan Key Laboratory of Southern Medicine Utilization, Yunnan Branch Institute of Medicinal Plant Development, Chinese Academy of Medical Sciences, Jinghong, China

**Keywords:** *Panax notoginseng*, identification, TaqMan probe, quantitative real-time PCR, traditional Chinese patent medicine

## Abstract

**Background:** There has been global concern about the safety and accuracy of traditional Chinese patent medicines (TCPMs). *Panax notoginseng*, also known as sanqi, is an important constituent of TCPMs. However, identifying the species contained in TCPMs is challenging due to the presence of multiple ingredients and the use of various preparation processes.

**Objective:** To detect *P. notoginseng* in TCPMs.

**Methods:** A TaqMan probe-based qPCR assay was constructed and validated with DNA extracted from *P. notoginseng* and adulterants. In total, 75 samples derived from 25 batches of TCPMs were tested using the constructed qPCR method.

**Results:** A TaqMan probe-based qPCR assay targeting *P. notoginseng* was established. The constructed qPCR assay could specifically discriminate *P. notoginseng* from *Panax ginseng*, *Panax quinquefolium* and *Curcuma aromatica* Salisb. cv. Wenyujin. The sensitivity study showed that the detectable DNA template concentration of *P. notoginseng* for this qPCR assay was 0.001 ng/μl. All 75 samples from TCPMs were confirmed to contain *P. notoginseng* by the qPCR assay.

**Conclusions:** The qPCR method can accurately identify *P. notoginseng* in TCPMs and is promising as a powerful tool for quality control and market regulation.

## Introduction

The roots and rhizomes of *Panax notoginseng* have been used as the traditional Chinese medicine (TCM) “sanqi” for many years and have been recorded in the Pharmacopoeia of the People’s Republic of China. Because of the obvious treatment effect of “sanqi” on many diseases including diabetes, inflammation and cardiovascular and cerebrovascular diseases (Liu et al., 2014; Zhou et al., 2017; Zhou et al., 2019; Xie et al., 2020), it has been used as an important component of traditional Chinese patent medicines (TCPMs). Therefore, TCPMs that contain *P. notoginseng* occupy a large share of the drug market. Quality control is the focus of TCPMs because they are directly applied in the clinic, affecting the safety of human health. Whether accurate raw materials are used is the origin of quality control for TCPMs because adverse drug reactions may occur if adulterants are used, as observed in the world-famous “aristolochic acid nephropathy (AAN)” incident, which was caused by the confusion of “Guan Mutong” with “Mutong”. However, the establishment of species identity in TCPMs is a great challenge. First, most TCPMs contain more than one species, and the ingredients in the preparation may interfere with each other. For example, the test items in the pharmacopoeia, such as macroscopic and chemical characteristics, are similar in some TCMs. Thus, advanced techniques targeting chemical markers may fail to identify species contained in TCPMs. Based on DNA sequences, molecular methods have played critical roles in the identification of TCMs in recent years with the advantage of identifying TCMs at the species level (Chen et al., 2014). Nevertheless, it remains difficult to identify the ingredients in TCPMs by these techniques because DNA may degrade after processing. Therefore, such a method is urgently required. Quantitative real-time PCR (qPCR) technology has been widely used in gene expression analysis, environmental monitoring and medication diagnosis due to its specificity, sensitivity and simple operation. Compared to other qPCR assays, TaqMan probe-based assays are more specific because the probe only binds to the complementary sequence and then produce a fluorescent signal. During the diagnosis, prevention and control of COVID-19, caused by novel coronavirus 2019, TaqMan probe-based qPCR technology has played a crucial role in nucleic acid testing and has been deemed the “gold standard” for COVID-19 diagnosis (Esbin et al., 2020). This study developed and validated a new qPCR assay based on a TaqMan probe to authenticate *P. notoginseng* and distinguish it from its closely related species *Panax ginseng* and *Panax quinquefolium* and the common adulterant *Curcuma aromatica* Salisb. cv. Wenyujin. Moreover, 75 samples of TCPMs (in the form of powder, capsules, tablets and suppositories) that should contain *P. notoginseng* were tested using our qPCR assay. This study showed that TaqMan probe-based qPCR is a powerful tool for the rapid authentication of *P. notoginseng* and the regulation of TCMs.

## Materials and Methods

### Materials

**FIGURE 1:**
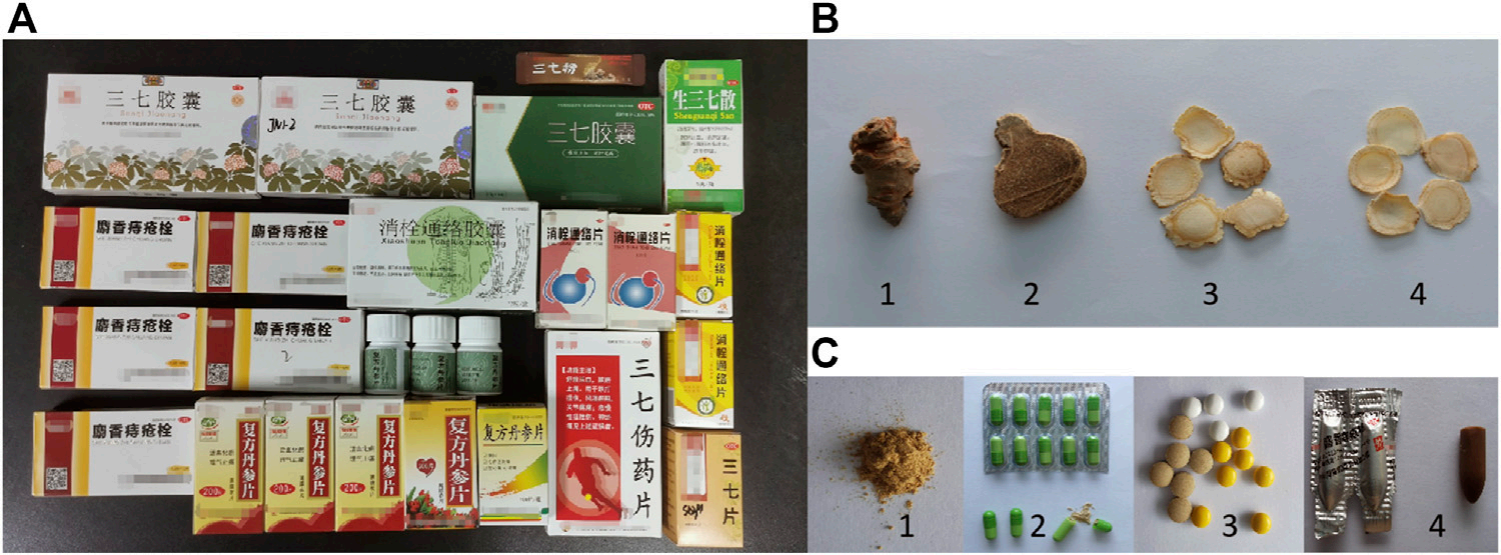
The samples used in this study. **(A)** 25 batches of traditional Chinese patent medicines; **(B)** Four samples of Chinese materia medicas:1) *Panax notoginseng*; 2) *Curcuma aromatica* Salisb. cv. Wenyujin; 3) *Panax ginseng*; 4) *Panax quinquefolium*; **(C)** Different dosage forms of traditional Chinese patent medicines: 1) Powder; 2) Capsule; 3) Tablet; 4) Suppository.

Four Chinese materia medica (CMM) samples of *P. notoginseng*, *P. ginseng*, *P. quinquefolium* and *C. aromatica* cv. wenyujin were collected from pharmacies (Figure 1). To ensure the accuracy of the CMM samples, DNA barcoding technology was used for authentication. In total, 75 samples derived from 25 batches of TCPMs from different manufacturers belonging to different lot numbers were collected from pharmacies (Figure 1; Table 1). According to the pharmacopoeia and description, all these TCPMs should contain *P. notoginsen*g powder. Among them, 18 samples contained only *P. notoginseng* as raw materials, 24 samples contained 3 CMMs, 18 samples contained 8 CMMs, and 15 samples contained more than 10 CMMs. All voucher specimens were deposited at the Institute of Medicinal Plant Development, Beijing, China.

**TABLE 1:** The information of traditional Chinese patent medicine samples.

Dosage form	Batch	Origin	Lot no.	Sample
Powder	Sanqi Fen	Beijing	20190102	3
	Sheng Sanqi San	Guangxi	200506	3
Capsule	Xiaoshuan Tongluo Jiaonang	Liaoning	201003	3
	Sanqi Jiaonang	Yunnan	1906040	3
		Yunnan	2008022	3
		Hangzhou	200601	3
Tablet	Sanqi Pian	Beijing	20120395	3
	Sanqi Shangyao Pian	Jilin	20200820	3
	Fufang Danshen Pian	Guangzhou	F20A011	3
		Guangzhou	C21A004	3
		Guangzhou	F18A026	3
		Beijing	20120923	3
		Beijing	20120476	3
		Beijing	20120862	3
		Shanghai	2010114	3
		Guangzhou	3200802	3
	Xiaoshuan Tongluo Pian	Beijing	20121172	3
		Beijing	19121180	3
		Heilong Jiang	171001	3
		Heilong Jiang	180302	3
Suppository	Shexiang Zhichuang Shuan	Hubei	210519	3
		Hubei	200603	3
		Hubei	2004003	3
		Hubei	2006005	3
		Hubei	200110	3
Total				75

### Methods

#### DNA Barcoding Identification

##### DNA Extraction and PCR Amplification

Total genomic DNA of CMMs was extracted using a plant genomic DNA extraction kit (Tiangen Biotech Co., Beijing, China) according to the manufacturer’s instructions. Total genomic DNA of TCPMs was extracted in the same way by adding a preprocessing step to rinse with nucleic acid separation solution three times (Xin et al., 2018a). Different pretreatment methods were adopted according to the dosage forms of TCPMs. DNA concentration and purity were determined using a Nanodrop 2000 (Thermo Fisher Scientific Inc., United States).

The amplification of the internal transcribed spacer 2 (ITS2) of nuclear ribosomal genes was performed by the universal primer pair ITS2 2F/3R (2.5 μmol/L) (Table 1). PCRs were performed in 25-µl volumes containing 2 µl of DNA, 12.5 µl of 2x Taq MasterMix (AidLab Biotechnologies Co., Ltd., China), 1 µl of each primer and 8.5 µl of distilled deionized water. The reaction conditions were as follows: 94°C for 5 min; 40 cycles at 94°C for 30 s, 56°C for 30 s, 72°C for 45 s and 72°C for 10 min (Chen et al., 2010).

##### Sequencing and Data Analyses

The purified PCR products were sequenced bidirectionally using Sanger sequencing. Contig assembly and the generation of consensus sequences were performed using CodonCode Aligner. Low-quality sequence data and primer sequences were removed. The BLAST method was used to identify species through the DNA barcoding database for traditional medicines (http://www.tcmbarcode.cn/en/).

### Primers and TaqMan Probe Design

The ITS2 sequences of *P. notoginseng*, *P. ginseng*, *P. quinquefolium*, and *C. aromatica* cv. wenyujin were downloaded from the DNA barcoding database for traditional medicines and our previous studies (Chen et al., 2014; Liu et al., 2016) and were then aligned using MEGA 6.0 software. Variable sites were identified by sequence alignment. The specific primers SQ3F/3R were designed based on the conserved region of *P. notoginseng,* and the probe was designed based on the variable sites (Table 2). The probe was labelled with 5′-carboxyfluorescein (FAM) and 3′-fluorescent quencher dye (BHQ1) for quantitative real-time PCR. The primers were synthesized by Sinogenomax Co., Beijing, China, and the probe was synthesized by Genewiz Biotech Co., Jiangsu, China.

**TABLE 2:** The sequences of primers and probe used in this study.

	Sequence (5′-3′)	References
ITS2 2F	ATG​CGA​TAC​TTG​GTG​TGA​AT	Chen et al. (2010)
ITS2 3R	GAC​GCT​TCT​CCA​GAC​TAC​AAT	Chen et al. (2010)
SQ 3F	ATT​AGG​CCG​AGG​GCA​CGT​CT	This study
SQ 3R	GCATTTGGGCCAACCGCG	This study
Probe	ATC​ATT​CCC​TCG​CGG​GAG​TCG​ATG​C	This study

### Quantitative Real-Time PCR

qPCR was performed in a 20-μl reaction system containing 10 μl of Probe qPCR Mix (2x) (Takara Biotech Co., Japan), 0.4 μl of each qPCR primer (10 μM), 0.8 μl of probe (10 μM), 2 μl of DNA and 6.4 μl of distilled deionized water. The qPCR conditions were as follows: 95°C for 30 s, 40 cycles at 95°C for 5 s and 60°C for 30 s (Bio–Rad, Inc., United States).

### Specificity, Sensitivity and Repeatability Testing

To validate the specificity of the TaqMan probe and primers, positive samples of DNA extracted from *P. notoginseng*, negative control DNA extracted from *P. ginseng*, *P. quinquefolium*, and *C. aromatica* cv. wenyujin and blank control distilled deionized water were detected synchronously. To determine the sensitivity of the qPCR assay, a series of tenfold dilutions (100 ng/μl-0.00001 ng/μl) of reference DNA (DNA extracted from *P. notoginseng*) were tested under the same conditions. Distilled deionized water was used as a negative control. All experiments were repeated three times to validate the repeatability.

### Detection of *P. notoginseng* in Traditional Chinese Patent Medicines

In total, 75 DNA samples extracted from 25 batches of TCPMs in four dosage forms that should contain *P. notoginseng* were tested using a TaqMan probe-based qPCR procedure. To ensure the consistency of the experiment, the concentrations of all the DNA samples were diluted to approximately 50 ng/μl. Distilled deionized water was tested simultaneously as a negative control. All experiments were performed under the same conditions in triplicate.

### Verification of Suspicious Samples by Clone Sequencing

Suspicious samples were amplified using specific primers of SQ3F/3R with the same PCR conditions of DNA barcoding identification. PCR products were purified with the Universal DNA Purification Kit (Tiangen Biotech Co., China) and then cloned into the pMD18-T vector and transformed into *E. coli* cells. Recombinant *E. coli* cells were cultured in Luria–Bertani (LB) liquid medium with 50 μg/ml ampicillin and then sequenced bidirectionally using Sanger sequencing.

## Results

### DNA Barcoding Identification

A total of 87 DNA samples were obtained, including 12 samples from four CMMs and 75 samples from 25 TCPMs. The concentrations of DNA samples extracted from CMMs were approximately 62.4–203.1 ng/μl. The ITS2 sequences of all the CMMs were successfully amplified, and the BLAST results showed that they were all derived from the correct species of origin. The concentrations of DNA samples extracted from TCPMs varied from 31.0 to 419.9 ng/μl. All the samples extracted from TCPMs were successfully amplified by ITS2 universal primers and sequenced except for the three samples extracted from “Sheng Sanqi San”. The concentrations of the failed amplified samples were 51.2, 57.3, and 52.9 ng/μl. The BLAST results showed that all the successfully amplified samples contained genuine *P. notoginseng*.

### Specificity, Sensitivity and Repeatability of the qPCR Assay

**FIGURE 2:**
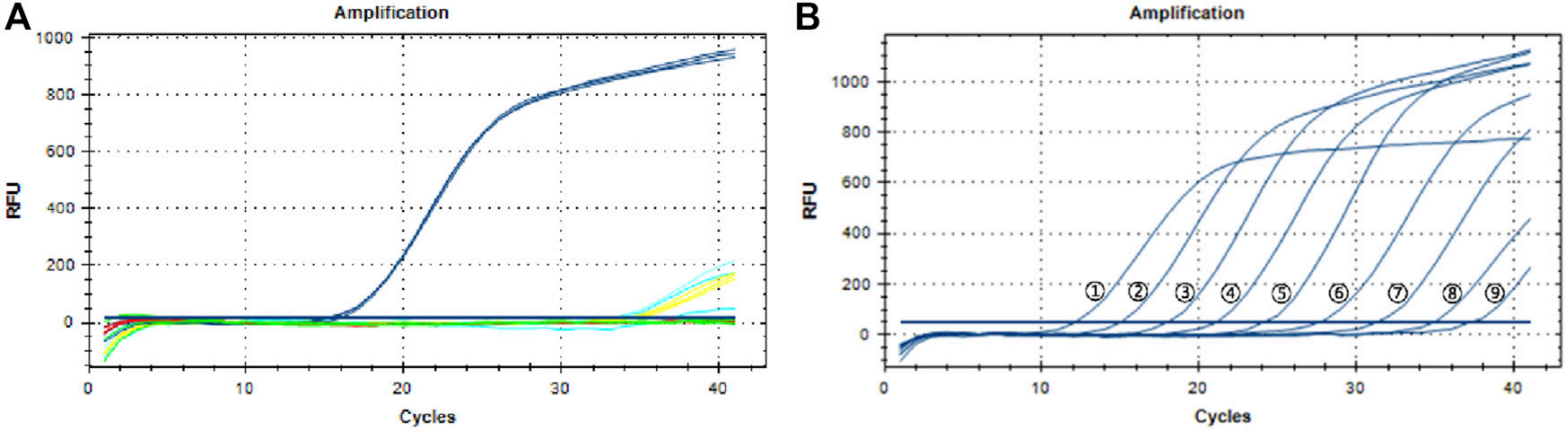
The specificity and sensitivity of the TaqMan probe based quantitative real-time PCR assay. **(A)** The specificity of the qPCR assay. Blue: Three samples of *Panax notoginseng*; Wathet: Three samples of *Curcuma aromatica* Salisb. cv. Wenyujin; Red: Three samples of *Panax ginseng*; Green: Three samples of *Panax quinquefolium*; Yellow: Blank control. **(B)** The sensitivity of the TaqMan probe based quantitative real-time PCR assay. Line①–⑧: DNA concentrations were 100, 10, 1, 0.1, 0.01, 0.001, 0.0001, 0.00001 ng/μl, respectively; Line ⑨: Negative control.

As shown in Figure 2A, the primers and TaqMan probe could distinguish *P. notoginseng* from *P. ginseng*, *P. quinquefolium* and *C. aromatica* cv. wenyujin. The curves of *P. notoginseng* showed a typical “S” shape. Extremely weak fluorescent signals of *C. aromatica* cv. wenyujin and the blank control were detected when Ct > 30, and no fluorescent signals of *P. ginseng* and *P. quinquefolium* were detected. These results indicated the high specificity of this qPCR assay. Figure 2B shows that this TaqMan probe-based qPCR assay displayed a better linear detection range. Good specific fluorescence curves were produced when the DNA concentrations were 100–0.001 ng/μl, and the Ct values were less than 30. Fluorescent signals were detected when the DNA concentrations were 0.0001–0.00001 ng/μl, but the curves did not show a typical “S” shape, and the Ct values were more than 30. Repeated experimental results showed high repeatability.

### Detection of *P. notoginseng* in Traditional Chinese Patent Medicines

**FIGURE 3:**
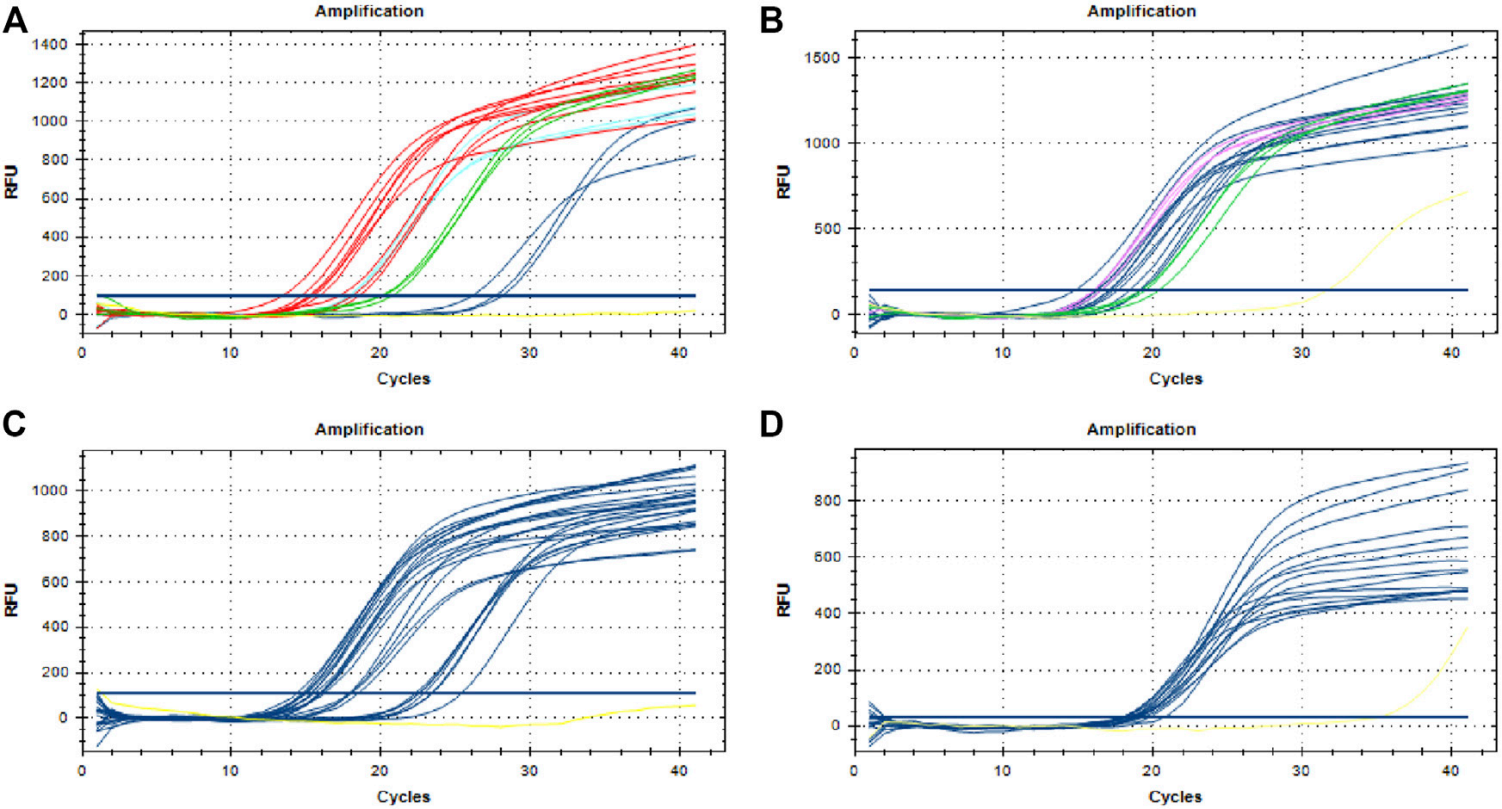
Detection of Panax notoginseng in traditional Chinese patent medicines. **(A)** Three samples of Sanqi Fen (whathet), three samples of Sheng Sanqi San (blue), nine samples of Sanqi Jiaonang (red) and three samples of Xiaoshuan Tongluo Jiaonang (green); **(B)** Three samples of Sanqi Pian (Rosered), three samples of Sanqi Shangyao Pian (green) and 12 samples of Xiaoshuan Tongluo Pian (blue); **(C)** 21 samples of Fufang Danshen Pian (blue); **(D)** 15 samples of Shexiang Zhichuang Shuan (blue); Yellow lines in all the pictures represent negative control.

A total of 75 DNA samples extracted from 25 batches of TCPMs were tested. Figure 3 shows that fluorescent signals were detected in all the samples, including the three samples that failed to be identified by DNA barcoding technology, which meant that all the TCPMs collected contained *P. notoginseng*. The results of the three samples derived from “Sheng Sanqi San” were different between DNA barcoding identification and the TaqMan probe-based qPCR. Thus, we cloned the target sequences of the three suspicious ones and sequenced them. The BLAST results showed that all three samples contained genuine *P. notoginseng*, which is consistent with the qPCR results. Our data validated the capacity of this method to be used in the identification of botanical species sources of herbal materials in TCPMs.

## Discussion

### TaqMan Probe-Based Quantitative Real-Time PCR Applied to the Identification of Plants in the *Panax* Genus Allows the Discrimination of Species With Close Relationships

TaqMan probe-based quantitative real-time PCR has been extensively used in recent years. Because of its high sensitivity and accuracy, this technique has played an important role in the detection of viruses, bacteria and pathogens, contributing to clinical diagnosis and plant quarantine (Pan et al., 2020; Katz et al., 2021; Panno et al., 2021). However, there have been limited reports about its application in the identification of medicinal plants. In this study, for the first time, we developed and validated a novel qPCR assay based on a TaqMan probe to detect *P. notoginseng*, a species in the *Panax* genus, as one of the most commonly used TCMs*.* The primers and probe were designed targeting the ITS2 sequence because research validated its discrimination ability in more than 6600 plant samples (Chen et al., 2010). Plants in the *Panax* genus showed highly similar DNA sequences, and only seven variation sites were found by comparing the ITS2 sequences among *P. notoginseng*, *P. ginseng*, and *P. quinquefolium*. In silico study indicated that the sequence of the probe can be only identified in *P. notoginseng*, which provided a theoretical support for the specificity of the probe used in this study (Supplementary Figure S1). Besides, in total 12 DNA samples extracted from four CMMs, including *P. notoginseng*, *P. ginseng*, *P. quinquefolium*, and *C. aromatica* cv. wenyujin, were used to establish and inspect this assay. The results indicated that it can specifically detect *P. notoginseng* from the closely related species *P. ginseng* and *P. quinquefolium* and the common adulterant *C. aromatica* cv. wenyujin. Previous studies revealed that the limit of detection (LOD) of qPCR assays varied from species to species. For example, the LOD of a multiple qPCR assay for bovine, porcine and fish DNA was 0.005 ng/μl, and that of a qPCR assay to detect *Cocos nucifera* was 0.001 ng/μl (Sultana et al., 2020; Wrage et al., 2020). The LOD of the qPCR assay constructed in this study was 0.001 ng/μl, which was highly sensitive. Therefore, this study proved the capacity of TaqMan probe-based qPCR to discriminate species in close relationships with similar DNA sequences, which hopefully overcomes the difficulty of discriminating closely related species.

### Feasibility of qPCR Application in the Quality Control of Traditional Chinese Patent Medicines

The safety and accuracy of TCPMs are subjects of global concern, leading to the requirement of efficient methods to identify the ingredients contained in TCPMs. Given the multiple ingredients and preparation technologies of different dosage forms, it is difficult for common molecular techniques to identify the species contained due to DNA damage and degradation. Some researchers have used high-throughput sequencing to monitor the species contained in TCPMs (Jia et al., 2017; Xin et al., 2018a; Xin et al., 2018b; Lo and Shaw, 2019), although the operation procedures were complex and time-consuming; thus, a simple, accurate and fast method was welcomed. In this study, a total of 75 DNA samples from 25 batches of TCPMs were successfully tested using the qPCR assay, proving its capacity to be applied in mixed and processed systems. All the samples tested in this study included four dosage forms, given that special preparation methods were applied before DNA extraction: 1) coatings of tablets were scraped off using a sterilized knife; 2) capsule shells were removed; and 3) suppositories were melted in a metal bath first. Fluorescent signals were detected in all the samples after appropriate pretreatments. The results were consistent with the DNA barcoding identification except for the three samples from “Sheng Sanqi San”. Further clone sequencing results of the three samples validated the existence of *P. notoginseng*, which was consistent with the qPCR results and verified the accuracy of the TaqMan probe-based qPCR. Because all the traditional Chinese patent medicines samples were collected from markets and produced by different manufactures, the quality of DNA extracted from them varied in both concentration and fragment length. As shown in Supplementary Figure S2, DNA extracted from traditional Chinese patent medicines samples degraded heavily. However, there are some long fragments clearly existed in DNA from “Fufang Danshen Pian” (DNA concentration of which was 31.0 ng/μl), while rare long fragments retained in DNA from “Sheng Sanqi San” (DNA concentrations of which were 51.2, 57.3 and 52.9 ng/μl). Therefore, the amplification of full length ITS2 barcodes from “Sheng Sanqi San” failed, and that from “Fufang Danshen Pian” succeeded. Furthermore, TaqMan probe-based qPCR assay targets a shorter sequence, it still works even though the DNA degraded. All these results demonstrated this technology could be applied to monitor the ingredients in TCPMs, contributing to the quality control of TCPMs.

### Quantitative Real-Time PCR Technology as a Powerful Tool for the Regulation of Traditional Chinese Medicines

Based on thousands of years of experience in health preservation and cutting-edge technologies, a number of achievements have been obtained in TCM research. Currently, TCM has attracted increasing attention worldwide, especially as TCM has played an important role in the treatment of COVID-19, greatly protecting human health (Huang et al., 2021). With this increased attention, strict pharmacovigilance is important. For TCM, quality control is the top priority. Useful regulation tools are required to control the safety and accuracy of TCM. With the rapid development of herbgenomics (Xin et al., 2019), molecular methods have gained attention due to the advantage of reflecting the genomic information, contributing to ensuring an accurate source. Technologies such as DNA barcoding (Chen et al., 2013), mini-barcodes (Diaz-Silveira et al., 2021) and nucleotide signatures (Liao et al., 2015) have been widely used to identify TCM species. However, these methods, which are based on sequencing, are time-consuming. Loop-mediated isothermal amplification (Zhao et al., 2016; Yang et al., 2019), which does not require sequencing, has also been applied for authentication, although several primers are required. Compared to other quality control methods, the qPCR assay constructed in this study has some advantages: 1) it has great specificity, accuracy and sensitivity; 2) it can be applied in TCPMs, can identify the ingredients at a species level, and can determine whether proper processing methods are used in the production of TCPMs because in some cases, extracts are used to produce drugs when the pharmacopoeia states that raw powder must be used; and 3) the operation of qPCR is very simple and rapid because no sequencing procedure is required, making this method suitable for market inspection. Therefore, it is considered to be a powerful tool for the regulation of TCM, ensuring safety and accuracy and accelerating the modernization of TCM.

## Data Availability

The sequence data reported in this paper have been deposited in GenBank under the accession number ON032876-ON032902.
