# Supplementary material for: TaqMan Probe-Based Quantitative Real-Time PCR to Detect Panax notoginseng in Traditional Chinese Patent Medicines
Source: Front Pharmacol. 2022 May 24;13:828948. doi: 10.3389/fphar.2022.828948 (PMC9171072; doi:10.3389/fphar.2022.828948)
Supplement: Supplementary file 1 [file DataSheet1.docx]

Supplementary Material

# Supplementary Table

Supplementary Table 1 GenBank accession numbers of the samples used in this study

| Sample Name | Sample ID | Accession Number |
| --- | --- | --- |
| *Panax quinquefolium* | PN0001MT01 | ON032876 |
| *Panax notoginseng* | PN0001MT02 | ON032877 |
| *Panax ginseng* | PN0001MT03 | ON032878 |
| Fufang Danshen Pian | ZCY0001MT01 | ON032879 |
| Fufang Danshen Pian | ZCY0001MT02 | ON032880 |
| Fufang Danshen Pian | ZCY0001MT03 | ON032881 |
| Sanqi Shangyao Pian | ZCY0001MT04 | ON032882 |
| Sanqi Fen | ZCY0001MT05 | ON032883 |
| Xiaoshuan Tongluo Pian | ZCY0001MT06 | ON032884 |
| Sanqi Pian | ZCY0001MT07 | ON032885 |
| Shexiang Zhichuang Shuan | ZCY0001MT08 | ON032886 |
| Xiaoshuan Tongluo Pian | ZCY0001MT09 | ON032887 |
| Sanqi Jiaonang | ZCY0001MT10 | ON032888 |
| Sanqi Jiaonang | ZCY0001MT11 | ON032889 |
| Fufang Danshen Pian | ZCY0001MT12 | ON032890 |
| Fufang Danshen Pian | ZCY0001MT13 | ON032891 |
| Shexiang Zhichuang Shuan | ZCY0001MT14 | ON032892 |
| Shexiang Zhichuang Shuan | ZCY0001MT15 | ON032893 |
| Shexiang Zhichuang Shuan | ZCY0001MT16 | ON032894 |
| Xiaoshuan Tongluo Pian | ZCY0001MT17 | ON032895 |
| Xiaoshuan Tongluo Jiaonang | ZCY0001MT18 | ON032896 |
| Xiaoshuan Tongluo Pian | ZCY0001MT19 | ON032897 |
| Sanqi Jiaonang | ZCY0001MT20 | ON032898 |
| Fufang Danshen Pian | ZCY0001MT21 | ON032899 |
| Fufang Danshen Pian | ZCY0001MT22 | ON032900 |
| Fufang Danshen Pian | ZCY0001MT23 | ON032901 |
| Shexiang Zhichuang Shuan | ZCY0001MT24 | ON032902 |

# Supplementary Figures


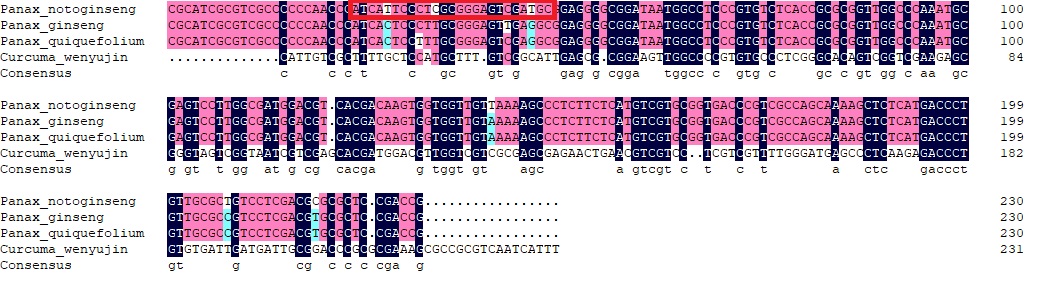


**Supplementary Figure 1.** Alignment of ITS2 sequences of *Panax notoginseng*, *Panax ginseng*, *Panax quinquefolium* and *Curcuma aromatica* Salisb. cv. Wenyujin.


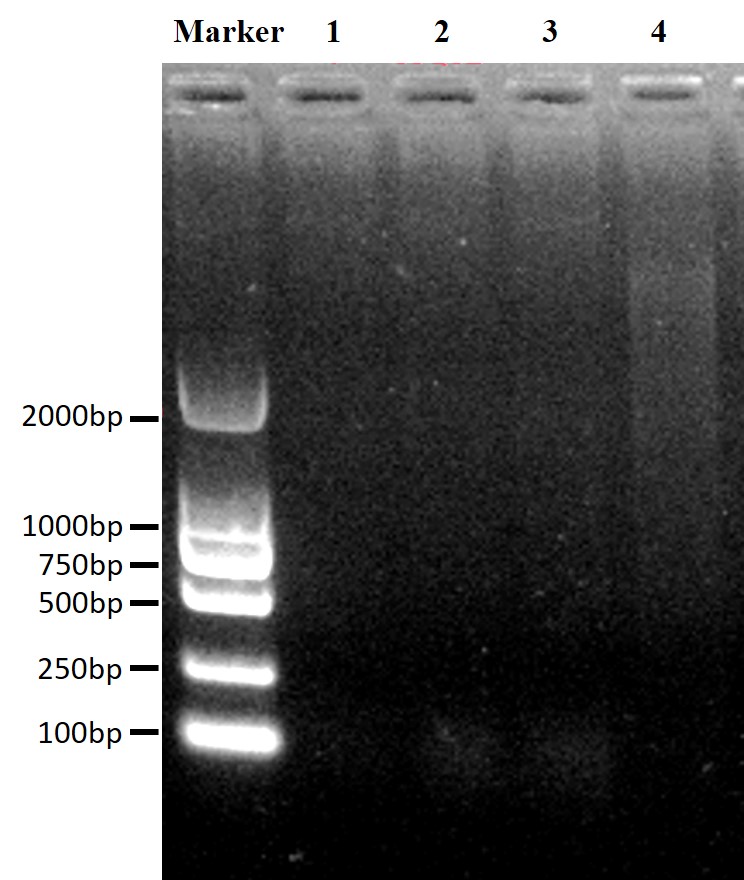


**Supplementary Figure 2.** Gel electrophoresis of DNA extracted from traditional Chinese patent medicines. Lane 1: Sheng Sanqi San (51.2 ng/μL); lane 2: Sheng Sanqi San (57.3 ng/μL); lane 3: Sheng Sanqi San (52.9 ng/μL); lane 4: Fufang Danshen Pian (31.0 ng/μL).
